# “When I feel well all over, I study and learn better” - experiences of good conditions for health and learning in schools in the Arctic region of Sweden

**DOI:** 10.1080/22423982.2020.1788339

**Published:** 2020-07-14

**Authors:** Catrine Kostenius, Lena Nyström

**Affiliations:** a Department of Health Sciences, Luleå University of Technology, Luleå, Sweden; b Norrbotten Association of Local Authorities, Luleå, Sweden

**Keywords:** Arctic region, health promotion, health literacy, education, learning, participatory, qualitative method

## Abstract

A challenge facing the Arctic region is the disengagement of both education and work among its youth. Only by supporting young people who are struggling with mental and physical health challenges can we begin to address this societal challenge. Education, mental health and social inclusion are prominent factors for future employment, income and independent living for young people. The aim of this study was to describe and understand the experiences of good conditions for health and learning in schools in the Arctic region. The 5-D appreciative inquiry method was used to explore 47 students’ and professionals’ experiences and future visions of their school. A phenomenological analysis resulted in three themes: “Standing as one”, “Having an apple a day”, and “Finding the end of the rainbow”. The findings revealed the necessity of promoting health and learning simultaneously in school and viewing health holistically. Health-promoting relationships permeate the findings of good conditions for health and learning. We argue for considering mandatory health education to increase students’ health literacy and making student participation and staff collaboration a priority in schools in the Arctic region. These findings, their practical implications, and future research directions are discussed.

## Introduction

A challenge facing the Arctic region is the disengagement of both education and work among its youth. Only by supporting young people who are struggling with mental and physical health challenges can we begin to address this societal challenge. Education, mental health and social inclusion are prominent factors for future employment, income and independent living for young people [1]. Although health is not the first aim of education, it is a factor that affects learning [2]. Because preventing health problems and promoting good health are associated with improved academic results, the integration of health into the educational arena warrants further attention [3]. The literature suggests that a holistic educational environment, where health promotion, health literacy, and a focus on equity between students and teachers are aligned, fosters good health and, thus, good learning [4–6].

Towards promoting health in the educational arena, school staff can use a disease-prevention approach that focuses on risks, challenges and disparities identifying problems and potential solutions. Another option is a health-promotion perspective, described in the World Health Organisation’s Ottawa Charter [7]. This approach entails making a conscious choice to explore what is well, the possibilities instead of the problems, and finding strengths and positive assets [8]. Exploring the good does not mean overlooking or disregarding the bad but, rather, it enables solving problems by amplifying the good [9]. Health-promoting schools (HPSs) have been high on the agenda worldwide since the WHO convened an expert committee on comprehensive school health education and promotion in 1995. Since then, much has been learned about successfully promoting health in schools, for example, the importance of clear leadership, setting well-defined goals, having high expectations of the students, fully involving students in the life of the school, and creating a social climate and environment that students appreciate [10].

The Arctic regional context has been taken into consideration in research and reports describing healthy schools in Canada, Finland, Norway, and Sweden that highlight what makes good, equitable, and healthy schools possible. Studies point towards a holistic approach where equity between students and teachers helps to provide a nurturing environment for good health and thus good learning. For example, findings from an HPS initiative in rural Nova Scotia, Canada suggest a supportive school ethos with positive teacher-student relationships enables student well-being in school [11]. The Swedish Schools Act regulates the mandatory work with health promotion in such a way that it permeates the school curriculum and involves the whole school [12]. Examples from Finland also suggest that an open school community is needed in which everyone has an equal voice where ethical questions need to be addressed consciously and systematically from early on [13]. The lessons from HPSs learned in Finland can be summed up in a well-balanced curriculum taught by proficient educators, time to play regularly, and a low-stress atmosphere in a learning environment that promotes student well-being [14]. Insights from Norwegian HPSs include that collaboration is key at the organisational level to facilitate the implementation of an HPS’s mission [15]. The need for gender-specific interventions has been suggested by both Norwegian and Swedish researchers recognising the early adolescence struggles with health problems in young males and females from remote areas [16,17].

According to Swedish school law and the national curriculum, the school should promote learning and harmonious development for all students while paying attention to health and lifestyle issues [18: 19, 20]. Student health care staff (i.e., school nurses, psychologists, and social workers) and teachers alike share the responsibility of promoting students’ health [21]. Collaboration and participation should extend beyond the professionals in school, as students need to be equally involved in both learning and health promotion processes [22]. This line of reasoning connects health and learning with the concept of health literacy, which entails people’s knowledge, motivation, and competencies to access, understand, appraise, and apply health information to making judgements and decisions in everyday life concerning health care, disease prevention, and health promotion to maintain or improve their quality of life [23]. To increase young peoples’ knowledge about health, the subject of health needs to be included in the school curriculum as a strategy to build health literacy and must be viewed as a part of a lifelong learning process that starts at a young age [24]. The aim of this study was to describe and understand the experiences of good conditions for health and learning in schools in the Arctic region.

## Methods

The authors have used appreciative inquiry (AI) which involves asking positive questions and using the answers as a starting point for development [9]. An appreciative process is about seeing the best in people, valuing activities, or qualities in organisations [25]. The specific AI process chosen for this study, the 5-D method, contains five steps: Define, Discovery, Dream, Design, and Destiny [26].

This method has been recommended as a strength-based model to improve school climate [27]. The 5-D method is a multimodal approach helpful for facilitating students’ exploration of school experiences [28]. An earlier version of the 5-D method, the AI 4-D cycle, was used to capture teachers’ experiences and perspectives about positive school development [29].

### Data collection

The participants were divided into small groups of 4–5 that were balanced regarding age, gender, profession, and nationality. The data collection process included:

1) Define: A topic of inquiry (health and learning in HPS development) was selected by the principal and school board and then communicated to the two authors. 2) Discovery: The participants were asked to consider examples of when health and learning intertwine at school and discuss why these situations were valuable and important. 3) Dream: Participants formulated visions and document future positive scenarios when health and learning go hand in hand. 4) Design: The participants planned out potential HPS development strategies by setting goals and suggesting new school activities and improvements. Each participant was asked to take notes about their experiences and then discuss these and responses to steps 2 and 3 in their small group. 5) Destiny: Finally, the participants cut and pasted pictures and written text from magazines and onto poster boards (1 per group) to visually represent a future in which their formulated goals and activities (step 4: design) align with their visions and positive scenarios (step 3: dream).

### Context and participants

This study was carried out in one municipality in the county of Norrbotten, located in the Arctic region of Sweden (latitude ~67º N, longitude ~20º E). The annual school evaluation 2016 indicated students’ health and academic achievement were decreasing. From 2017 to 2019, a research group that included the two authors was charged by the municipality with developing activities to promote student health and learning and collecting data for research studies. The present study was undertaken in the first year of this project to document and examine students’ and professionals’ experiences and perspectives regarding conditions conducive for good health and learning.

All students and professionals in the municipality’s upper secondary schools were invited to participate, which, at the time, included approximately 150 students, 30 school staff, and 12 school politicians. A total of 47 participants, both students and professionals, took part in the study. The 27 students (20 males, 7 females) were in grades 7, 8, 9, and the introductory language program for young immigrants. The 20 professionals (4 males, 16 females) included teachers, teaching assistants, school nurses, principals, and politicians. The participants came from all three schools in the municipality, which has a large number of immigrants. This study included individuals from Afghanistan, Finland, Somalia, Sudan, Sweden, and Thailand. Due to the risk of identifying the municipality and the participants, we have refrained from supplying additional information.

### Ethical considerations

The municipality’s development manager distributed the invitations to participate to all students and professionals in the municipality’s upper secondary schools. The researchers provided oral and written information about the study to all potential participants, as outlined in the Helsinki Declaration [30]. Oral and written information about voluntary participation and confidentiality were also provided, in accordance with Swedish ethics laws [31]. All participants were over the age of 15 and participated willingly; nevertheless, an informative letter was given to the participants’ parents. The study was reviewed by the local ethics committee [2017/128-31] and all subjects provided written informed consent before their participation.

### Analysis

The analysis was based on a hermeneutical phenomenological approach to lived-experience research in three phases [32]. The participants’ written reflections and collages constituted the empirical data and represented “experiential narrative material for developing a richer and deeper understanding of a human phenomenon” [32, p. 66]. In the first phase, *reflectively appropriating*, we considered all of the data as a whole by reviewing the text and collages repeatedly to provide an overview of the participants’ lived experiences. The two authors coded the data based on their independent reflections and then compared and discussed these to create several codes reflecting their combined picture.

In the second phase, *clarifying*, the authors grouped the codes based on the similarities and differences among the data and used a mind map to determine how these codes were connected. In the third and final phase, *making explicit the structure of meaning of the lived experience*, the earlier phases of the analysis were used to create themes that explained the patterns within the data. Each of the three themes includes variations among the participants’ perspectives. Illustrative quotations were chosen to exemplify each theme, give voice to the participants, and enhance the study’s credibility [33]. No software was used in the analysis process, instead, the two authors took independent handwritten notes in phases 1 and 2 and used codes to communicate their understanding of the material. Finally, a mind map was drawn on a whiteboard when forming themes in phase 3.

### Methodological considerations

Participatory methods reflect empowerment which is a vital aspect of health promotion [7]. The 5-D appreciative inquiry method was used to explore the 47 participating students’ and professionals’ experiences, visions, and ideas for their school. Parents were not included although some of the participating adults were parents, which could be seen as a limitation. We are fully aware of parents’ unique position and important role in supporting health promotion efforts for students together with school staff [34, 35]. However, as the focus of this study was the school environment, the students and professionals directly affected in some way were our primary focus. This “power of everyone” approach can promote health literacy and positively affect learning [24,36].

According to the Swedish National Board of Health and Welfare and Swedish National Agency for Education, there is an assignment for teaching staff and student health professionals alike to cooperate with parents and guardians [21]. A unique constellation of students and professionals representative of multiple professions and countries of origin were invited to participate. The method and participants chosen are strengths of the present study as they reflect arguments that the whole school community should be given a voice and that inclusive dialogue supports positive action and enables progress [36]. Research involving researcher with both health and education backgrounds ensures co-creation in the evaluation of HPSs [37].

Therefore, another participation-associated strength of the present study is the experience of its two researchers. The professional background of the first author is in the field of child healthcare, and, academically, she is a professor of health science with a bachelor’s degree in pedagogy. The second author is an experienced secondary-school teacher with a master’s degree in education. Both authors have extensive experience with research and development projects during the past two decades.

## Results

The phenomenological analysis resulted in three themes: *Standing as one, Having an apple a day*, and *Finding the end of the rainbow*.

### Standing as one

The participants described conditions conducive for good health and learning with a clear connection to interpersonal interactions and relationships. Many mentioned physical space as an enabling factor. Activities promoting fellowship and solidarity were both educational and social and took place in a variety of spaces and contexts throughout the school day. One group wrote that “activities where students and teachers from different grades and mixed ages make something together” were health-promoting. Also, the participants mentioned learning from each other and sharing time and experiences in different activities, not only focusing on school subjects but also socialising. They appreciated spaces that provided opportunities to learn from each other as humans beyond profession and age. One group wrote, “Politicians, teachers, principals, and students can meet and talk.”

The participants emphasised the importance of creating relationships within the whole school, including the entire staff. The participants described activities where everyone was included and communication and socialising enabled health promotion. For example, the participants described situations when students shared their experiences of what is going on in school with people working in the administration office. One group highlighted the importance of “creating a sense of we.” Another group explained how inclusive activities “promote a great deal of dedication, many ideas, a feeling of equality, and collaborative learning.” The participants recommended that more time be allocated for sharing reflections between teachers and students and among students alone.

In addition to teachers, several other school professionals were identified as supportive, such as the school nurse, health coach, social worker, and principal. The participants stressed that the profession did not matter as much as the adult being present and engaged. One group specifically mentioned needing “an adult outside during recess” to feel safe. The participants described frequently enjoying dialogues with both students and professionals that focused on the positive aspects of health.

Having time to share was instrumental for the participants to increase learning, support personal development, and effectively solve problems. One group wrote, “Working in groups, talking with each other, and having discussions enable solutions.” The participants described activities that connected people and offered social opportunities, which included playing sports or games and conversing. One group stressed the importance of having time for health promotion during regular school hours, included in the regular lesson plan, and described activities such as conversations and reflections about current health topics. Another group described an activity as “talking about life, common everyday matters, creating togetherness and security.”

The student council was described as providing a democratic context in the school. The participants argued for increased student participation and a higher position of power in the school, through the student council and also within every classroom. One group wrote, “Take the students’ thoughts and opinions seriously and give them [the opportunity to] influence.” Within the classroom, participants mentioned peer review as an example of an activity that promotes health and learning. Another group wrote, “We all need appreciation … so the next time, you work harder.” However, it was not always about being active together. The participants describe sharing moments of silence and relaxation with other students as health-promoting.

The participants highlighted the teachers’ contribution to students’ health and learning. One group mentioned how important the teachers are for the students “by supporting and being nice to us.” The participants suggested designating a room for students to do their homework with support from teachers during and after the school day. The participating professionals described collegial support and a willingness to co-create. One group envisioned a future in which “teachers are happy when they come to school, share, learn, and cooperate with each other.” Another group wrote that they appreciated “ … when a student spontaneously comes up to you and helps out, when you have your hands full.” Taken together, the data consistently indicate that being surrounded by kind people supports good health and effective learning.

### Having an apple a day

According to the participants, conditions conducive to good health and effective learning include many activities, tools, places, and spaces that encourage health and learning opportunities. The reported activities included a variety of physical exercises, both invigorating and relaxing, such as walking, biking, dancing, and sports (e.g., ice hockey, floor hockey, and soccer). The participants mentioned a teacher who consistently takes 20-minute outdoor walks with the students before the day’s lessons, referred to as the “Maria walkabout.” The participants described this physical activity as social, invigorating, and positively impacting the students’ studies. One group wrote, “The Maria walkabout – we go outdoors in the refreshing air, which makes us more energetic and our brains work better.” Some physical activities were described as promoting both physical and mental well-being; for example, yoga, which consists of easy-to-learn body positions and breathing techniques that can reduce stress and anxiety. Another group wrote, “Micro breaks – yoga and movement during lessons.”

The participants experienced time as an important element of health-promoting activities. They described the practical execution of activities between lessons or as a part of mandatory lessons as a supportive measure to increase educational achievement. One group wrote about the importance of “relaxation in the schedule, time for peace and quiet.” The participants described relaxing activities, such as massages and longer breaks between lessons, or, as one group explained, the need for “an empty hour – taking the time for a peaceful moment.” However, some participants also asked for the opposite, namely shorter school days instead of “empty hours” in the schedule. One group suggested that spending less time in school is health-promoting: “School until 1 PM on Fridays ….” Another time-related aspect mentioned in this study was flexible hours or, as they called it, “flex-time,” that entails school days begin earlier or later based on the individual student’s optimal time of day for learning.

The participants also described tools to promote health. For example, physical health was enhanced by comfortable, ergonomically-tested chairs and gadgets to reduce stress and increase learning. One group wrote, “Students in grade 1 use balance balls as a way to increase concentration.” The participants described the need for practical activities that were within their subject areas and allowed for working with their hands. Another health-promoting aspect that the participants described was designating spaces where students, school staff, and politicians can meet. The participants suggested a physical room, like a café, where there is space to eat snacks and socialise. One group wrote, “The café has been worth gold – we hope it will be [open] in the future too.” Other physical rooms that were mentioned were a room in which to do homework. However, the participants also asked for the opposite describing a place to not work as one group wrote, “A room for rest and contemplation, designed with comfortable couches and cozy lighting … [with] a sign on the door – ‘Do not disturb! Relaxation room!’”

Additionally, clean showers and dressing rooms to use after physical education classes were noted as promoting health in school. The participants highlighted “nature” as a health-promoting aspect and suggested using nature as a place to play, relax, and learn. One group suggested “[placing] tables outdoors so we can work outside.” The data on including nature ranged from spending time outdoors during the school day to taking a trip to the mountains for extended time in nature.

Nutrition was mentioned by the participants as a valuable aspect of promoting health and learning. They described food as a tool and eating as an activity, which were connected to places in their school. In terms of eating breakfast together as part of the school day, having fruit available or healthy snacks for sale in the cafeteria was suggested. An “ice cream stand” was also on one group’s wish list. Food was not only a matter of nutrition but also described as a social activity and an opportunity to learn about a healthy lifestyle – for example, by connecting a healthy breakfast with academic achievement.

Actively choosing what food to eat was health-promoting according to the participants. One group wrote, “Food choices make you happy.” Happiness was connected to having a choice in general, as well as to learning. Another group wrote, “The students come to school happy, want to learn, and are included in decisions.” Similarly, one group wrote, “It is fun to learn.” The connection between health and learning was evident in the participants’ experiences. The participants described the sense of wholeness and their experiences of when health and learning were integrated. One group wrote, “When I feel well all over, I study and learn better.”

### Finding the end of the rainbow

The participants’ visionary thoughts about promoting health and learning included fundamental values of ethics permeating structures within the organisation. They described how ethical values are needed in all activities and approaches to building a health-promoting culture. According to the participants, important values included but were not limited to aspects of general human rights and, more specifically, children’s rights. They described student participation as a natural part of activities and approaches in their dream school. For example, the students co-created a school schedule with teachers, and one group gave voice to the principal: “The student council’s work is important to me as principal, taking students’ thoughts and opinions seriously, allowing students to truly participate and be able to have an influence.”

The participants described their school as a place where trust, security, and safety were not only encouraged but self-evident. One group envisioned that “everyone would feel safe at school and everyone would be seen.” The school of their dreams was described as an arena in which everyone’s attitudes enforced tolerance and where hopefulness and inclusion were fundamental values that were practiced daily. They described a safe place where people are accepted for who they are, making it more likely that they will dare to venture outside of their comfort zone, open up to cooperate with others, and learn from each other.

The participants described how openness to diversity is a prerequisite for having everyone feel comfortable and everyone’s responsibility. One group envisioned that “we feel well and show respect.” Another group suggested using the golden rule as a starting point, writing, “Do unto others as you would have them do unto you.” Supporting diversity was described in terms of mixing groups of professionals and students, boys and girls, and students from different countries and ages in activities as a way to learn about and from each other. One group wrote about “having a joint activity one day each term uniting students from all grades.” They stressed the importance of school being safe and inclusive. In their descriptions of the school of their dreams, appreciation, friendliness, and kindness were commonly practiced among school staff and students and also towards oneself; as one group wrote, “It is time for ‘self-kindness.’”

The notion of democracy in school was described and exemplified by the participants as genuine participation reflective of the students as important co-creators. They described opportunities for students to partake in not only daily work but in the long-term development within the school organisation. They described values that included respect for each other and responsibility for the shared space and encounters. The participants gave examples of fundamental values that were practiced within the school’s physical and social environment. They argued that student participation contributes valuably to creating the conditions necessary for promoting health and learning in school. One group described a future in which students were able to make decisions on things that they view as valuable, such as “organising groups of furniture in a creative fashion where we can share knowledge and learn together.” Another group envisioned student participation taking the form of “planning the school day, the current schoolwork, and activities together with the teachers.”

The participants connected learning to different school subjects that included maths and general learning strategies. According to the participants, knowledge of effective learning strategies provides a helpful understanding of how we learn. One group highlighted that “knowing about and sharing different strategies to learn is much appreciated.” The participants stressed the importance of taking control of their learning process by identifying individual short- and long-term goals connected to the curriculum. According to the participants, learning new strategies and sharing experiences with other students inside and outside of the classroom made the learning process easier, which resulted in a sense of well-being. One group remarked, “Working together in a group makes it easier.” The participants described fundamental values, including empowerment, appreciation, and sustainability as instrumental in creating conditions that encourage good health and learning. One group provided the following metaphor: “[We] wish that school were a greenhouse where we grew outstanding pumpkins, allowing everyone to succeed.”

The participants described school as a place where students develop life skills and embrace fundamental values that inform healthy choices throughout life. The participants described HPS development as the process of enabling conditions that promote good health and learning. Some suggestions were direct and without much preparation, such as allowing everyone to participate and become involved in for example planning health promoting activities. Other changes, such as fostering a school culture where trust, security, and safety are readily apparent, require more time. One group wrote, “Don’t be afraid of the slow progress but watch out for stagnancy.”

## Discussion

The findings revealed the necessity of promoting health and learning simultaneously in school and viewing health holistically. Health-promoting relationships permeate the findings of good conditions for health and learning. We argue for considering mandatory health education to increase students’ health literacy and making student participation and staff collaboration a priority in schools in the Arctic region.

These findings, their practical implications, and future research directions are discussed below.

### Promoting health and learning simultaneously and viewing health holistically

The findings point to the necessity of promoting health and learning simultaneously in school and viewing health holistically. According to the participants in this study a prerequisite for students reaching their educational goals while feeling well is that the students, staff, school leaders, and politicians understanding that school is an environment in which learning and health are intertwined. Interestingly, in line with previous studies, the participants described many places and spaces that are connected to their ability to learn, and their experiences suggest that physical, mental, social, and existential dimensions of health affect their learning [38, 39]. Viewing health holistically is in line with recommendations for effective and sustainable health promotion efforts [40]. This entails being aware of the physical, mental, social, and existential dimensions of health, which overlap [38] and impact learning [3]. The participants shared their perspectives of different dimensions of health. For example, in the sub-theme *Having an apple a day*, the participants described outdoor walkabouts that were experienced as social and invigorating, which is comparable to recent research that found that peers can offer paths to successfully promote physical activity [41]. Another example affecting the mental health of the participants was happiness, highlighted as a good condition for health and learning. In the theme *Having an apple a day*, situations resulting in feelings of happiness was connected to health. These included instances when the students were included in decisions, chose the school lunch menu, and when the students and teachers came to school feeling happy. These data support the discovery of happiness in education as crucial for effectively integrating health and social aspects, which includes the right to be treated equally and school being a place where people’s dignity is valued [42]. “Developing health in a school shows five components of health, including happy students, happy organisation, happy environment, happy family, and happy community, that become the scope of the term ‘school health’” (p. 520: [42]). It had been suggested that happiness should not only be an aim for education, but education should contribute to personal and collective happiness [43].

The theme *Finding the end of the rainbow* encompassed the participants’ vision of the future and revealed what they find meaningful, their hopes, and their optimism, which are all existential dimensions of health according to the WHO. The WHO described eight existential dimensions of health associated with high quality of life: spiritual connection, meaning and purpose in life, experience of awe and wonder, wholeness and integration, spiritual strength, inner peace, hope and optimism, and faith [44]. These eight aspects are a way to explore thoughts, actions, and feelings as humans relate to different life situations in relation to themselves, their context, and personal beliefs [45].

### Health-promoting relationships

Health-promoting relationships permeate all of the themes constructed in this study. The participants’ visions of good conditions for health and learning were clearly connected to interpersonal interactions and relationships that extend beyond age, profession, and physical space. This echoes the fundamental need for social interactions and meaningful encounters with others in school to strengthen the feeling of belonging, which is instrumental in promoting health among students [46]. Furthermore, in the themes *Having an apple a day* and *Finding the end of the rainbow*, the participants’ experiences and visions of their dream school consistently involved kindness in terms of friendliness, being nice, helping out, and being supportive. These findings reflect the notion of doing good for others and argument that what we teach in school today represents what we will see in tomorrow’s society [47]. Building a listening school environment is essential because relationships matter more than institutional systems [48].

In the themes *Standing as one* and *Having an apple a day*, the participants frequently mention maximising inclusivity. They report that activities that include everyone not only promote health and well-being but also strengthen the dedication to and actualisation of equality, which may likely increase creativity and collaborative learning. These findings revealed that health-promoting activities supported student learning in traditional school subjects, which can be compared to earlier studies [37]. They suggest that interventions including the whole school, not just isolated groups of students or professionals, make a difference in students’ health and learning. Furthermore, to build social connectedness in school, teachers need to allot time within the curriculum to develop the social and emotional well-being of their students, also noted in previous research [49]. A social-emotional school climate that encourages students’ social and emotional connections to their teachers support adolescents’ educational and career aspirations [50]. School is a multi-purpose institution that extends beyond academic goals and that we need to tend to students’ fundamental need for caring relations [51]. This is in line with a recent report from the Swedish National Agency for Education on preventing upper secondary school dropouts [52].

The agency recommends an inclusive and safe climate with positive relationships and a school culture that promotes learning. The theme *Standing as one* highlights the need for caring relations in school not only promotes students’ health and learning but is also positive for professionals – a win-win situation for everyone.

### Mandatory health education

Based on the findings of this study, to increase students’ health literacy, we suggest including health education as a mandatory subject or a distinct part of other subjects in Swedish school curriculums and making student participation and staff collaboration a priority in schools in the Arctic region. The concept of health literacy, as described by the World Health Organization [23], can be helpful when arguing for increasing students’ competencies to not only access, understand, appraise, and apply health information but to make judgements and decisions to promote their health and learning and improve quality of life. Health literacy is about learning an essential skill and ought to be put into the school curriculam, as strategies to build health literacy must be viewed as part of lifelong learning [53].

In Finland, the national school curriculum includes mandatory health education to increase students’ health literacy [54, 55]. This focus on health literacy within the educational arena could prevent health problems and ensure health promotion, which are linked with improved academic performance [3]. In the theme *Standing as one*, strategies for giving students a stronger voice included taking the students’ thoughts and opinions seriously. This was described as a context in which democracy is brought into practice, which would allow students to have an influence. This aligns with the arguments that an ethical and open school community in which everyone has an equal voice will enable health education to promote health literacy of [13].

In the envisioned dream school presented in the theme *Finding the end of the rainbow*, student participation was considered a natural part of school activities and educational approaches that echo both human and children’s rights. Similarly, the Universal Declaration of Human Rights [56] states that human rights apply to everyone “without distinction of any kind, such as race, colour, sex, language, religion, political or other opinion, national or social origin, property, birth or other” (Article 2, p. 2). Additionally, the Conventions of the Rights of the Child, which has been the law in Sweden since 1 January 2020 [57], states that children are entitled to have a say in decisions that affect them [58]. This same intention is found in Swedish curriculums, which state that student participation and influence should increase with age [19,20]. Therefore, it is not sufficient to communicate knowledge about democratic values, but working forms of these values need to be actively applied and consistently practiced in school.

### School staff prioritising student participation

The theme *Standing as one* points to students as co-creators who truly participate and influence the daily work of their school. This is similar to the arguments that there is a need for a new order of practice acknowledging the value of students’ active participation [59]. Schools need to be viewed from the students’ perspective by tuning into their experiences and perspectives and treating them as active participants in school improvements [53]. Similarly, there are recommendations that a democratic paradigm in HPSs develops students’ abilities to influence their lives and society [termed action competence] in solving problems and facilitating change [60]. Furthermore, these findings echo descriptions of ademocratic HPS where aparticipatory approach is practiced that involves students in both learning and health-promoting processes [22].

Taken together, data from the themes *Standing as one* and *Finding the end of the rainbow* emphasise that engaged teachers improve students’ social environment. Teachers’ roles in HPS development warrants further examination because teachers are the primary agents of change in academic environments but not experts in health promotion [61]. Teachers need both educational competence and high levels of action-oriented knowledge and insight to be effective educators [60]. Findings from Finnish and Norwegian schools in the European network of HPSs contradict the notion of teachers being primarily interested in teaching their respective subject matters (e.g., language, mathematics, history) and not caring about the health and wellbeing of their students [62,63]. Nonetheless, according to the findings of the present study, teachers play an integral role in fostering a positive school environment prioritising student participation.

### Collaboration among school staff

In the theme *Standing as one*, the participants described cooperation across professions towards promoting health and learning simultaneously in their school. Similarly, earlier research found that cooperation within the school and also between local schools facilitate the implementation of an HPS’s mission [15]. To educate and promote health and well-being simultaneously, these concepts need to be not only firmly embedded in educational policies but acted on in school practices and shared with the students and parents. Similarly, in a study of factors that support school attendance, the importance of teachers supporting students is described [64]. Collaborative relationships empower academic professionals to effect positive change in the health of the entire school community [65].

Unfortunately, previous research shows that the conditions that enable collaboration across occupations are lacking in Swedish schools today [66], Also, professional walls and silos encapsulate creativity and innovation and foster within-silo cultures that are resistant to penetration and change [67]. To overcome these hurdles, health promotion needs to be built on effective partnerships while “developing a science of cooperation” (p. 25) [65]. Health dialogue that are child-focused, systematic, and structured represent opportunities for collaboration among teachers, parents, school nurses, and other health professionals [68].

The theme *Finding the end of the rainbow* highlights the connection between health and learning, where fundamental values, such as democracy, equality, and inclusion, are practiced throughout the entire school organisation. Viewing health promotion as an effective way to improve educational outcomes will help to intertwine health and learning in school, as described in the Swedish national curriculum [19,20]. The findings of the present study focused primarily on the school as a physical place and creating spaces for interpersonal communication and relationship building. This calls for a holistic educational environment, where health promotion, health literacy, and focus on equity between students and teachers are aligned [4,5,6]. The findings revealed by this study consistently centre around the health-promoting factors of collaboration and participation, which fits with the whole-school approach [12]. Add that collaboration between stakeholders (i.e., students, teachers, parents, communities, researchers) is often challenging because their values, cultures, and traditions differ [10]. Nevertheless, the findings suggest the need for activities that encourage co-creation, regardless of profession and enable the expression of student voices and their participation in everyday schoolwork. However, this hinges on understanding the conditions required for successful collaboration across different occupations within school [65, 66]. Similarly, partnership and mutual understanding between the education and health sectors that build trust and capacity are needed to make HPSs successful within the national educational system [69].

### Practical implications and future research

The following three implications for practices illuminate the participants’ experiences of conditions that foster good health and learning in schools. These are connected to the Swedish national curriculum and founded in democracy to address health and lifestyle issues in school [19, 20].

1) First, for students to reach their educational goals while also feeling well, students, staff, school leaders, and parents must understand that school is where learning and health intertwine. To educate and promote health and well-being simultaneously, these concepts need to be firmly embedded in educational policies, acted on in school practices, and shared with students and parents.

2) The participants’ visions of the conditions conducive for good health and learning involved interpersonal interactions and relationships that transcend age, profession, and physical space. A school-wide commitment to practicing inclusion and participation, which are health-promoting factors, promotes impactful partnerships between students and staff. A mutual understanding between the education and health sectors builds trust and the capacity to make HPSs successful within a national educational system. More cooperation among school staff across professions in school and practice-based research on this topic are warranted.

3) Include health education as a single, mandatory subject or a distinct part of other subjects to increase health literacy. This focus on health literacy within the educational arena should work to prevent health problems and ensure health promotion, which are linked with improved academic performance.

Finally, for HPS interventions to improve young people’s social and emotional well-being, the interventions need to be effectively adopted and integrated into school practices and sustained over time [70]. Add that healthy policies, school’s physical environment, action competencies on healthy living, and community links have the most significant impact on a wide range of health-related outcomes that can help educators throughout their HPS journey [71]. However, the challenge of the sustainability of efforts promoting health and learning simultaneously requires further research. Although this study did not examine the sustainability of HPS interventions, the findings point to the necessity of promoting health and learning simultaneously and viewing health holistically suggesting mandatory health education, and making health-promoting relations, student participation, and staff collaboration a priority in schools in the Arctic region.
